# Therapeutic Efficacy of Human Monoclonal Antibodies against Andes Virus Infection in Syrian Hamsters

**DOI:** 10.3201/eid2710.210735

**Published:** 2021-10

**Authors:** Brandi N. Williamson, Joseph Prescott, Jose L. Garrido, Raymond A. Alvarez, Heinz Feldmann, Maria I. Barría

**Affiliations:** National Institutes of Health, National Institute of Allergy and Infectious Diseases, Hamilton, Montana, USA (B.N. Williamson, J. Prescott, H. Feldmann);; Robert Koch Institute, Berlin, Germany (J. Prescott);; Ichor Biologics LLC, New York, New York, USA (J.L. Garrido, R.A. Alvarez);; Universidad de Concepción, Concepción, Chile (J.L. Garrido, M.I. Barría);; Universidad San Sebastián, Puerto Montt, Chile (M.I. Barría)

**Keywords:** recombinant human monoclonal antibodies, Andes orthohantavirus, Syrian hamster, viruses, zoonoses

## Abstract

Andes virus, an orthohantavirus endemic to South America, causes severe hantavirus cardiopulmonary syndrome associated with human-to-human transmission. No approved treatments or vaccines against this virus are available. We show that a combined treatment with 2 monoclonal antibodies protected Syrian hamsters when administered at midstage or late-stage disease.

Hantavirus cardiopulmonary syndrome (HCPS) is a severe disease caused by infection with pathogenic New World orthohantaviruses, including Andes virus (ANDV). ANDV is transmitted to humans via the aerosolized excrement of the rodent reservoir and vector, the *Oligoryzomys longicaudatus* long-tailed pygmy rice rat (*1*). Unlike other orthohantaviruses, ANDV has been associated with human-to-human transmission and is associated with a high case fatality rate of 35%–40% (*2*–*5*). The large 2018 ANDV outbreak in the Austral Patagonian region at Epuyén village (Chubut Province, Argentina) reemphasized the probability of person-to-person transmission, highlighting the urgent need for specific therapies and vaccines (*5*).

ANDV infection has a long incubation period of 2–3 weeks, followed by a 3- to 5-day prodrome phase characterized by fever, headache and gastrointestinal symptoms. HCPS develops over a 2–7 day period characterized by falling blood pressure, lung edema or failure, cardiac shock, and death in a substantial number of patients (*6*). As of August 2021, treatment for HCPS remains supportive, requiring interventions such as oxygenation, mechanical ventilation, extracorporeal membrane oxygenation, and fluid balancing (*6*). No licensed specific treatment options or vaccine are available. In HCPS patients, high hantavirus-specific neutralizing antibodies correlate strongly with survival, milder disease outcomes, and faster recovery (*7*,*8*). Passive transfusion of specific monoclonal antibodies (mAbs) protected animals from ANDV challenge in the lethal Syrian hamster disease model (*9*–*11*). All of these data suggest an important role for neutralizing antibodies in controlling orthohantavirus infections in vivo.

Our group has previously reported that mAbs isolated from HCPS survivors (clones JL16 and MIB22) protected hamsters from lethal ANDV challenge when mAb treatment was initiated early postinfection, suggesting potent postexposure efficacy (*11*). We sought to develop a late-stage disease treatment schedule for mAb treatment in the Syrian hamster model of ANDV disease.

## The Study

The Syrian hamster model of lethal ANDV infection has been well established as the only animal model recapitulating many characteristics of HCPS (*12*,*13*). All infectious ANDV work was conducted in the Biosafety Level 4 facility at Rocky Mountain Laboratories (National Institutes for Health, National Institute of Allergy and Infectious Disease, Hamilton, MT, USA), according to standard operating protocols approved by the Institutional Biosafety Committee. All animal experiments were approved by the institutional Animal Care and Use Committee.

We inoculated hamsters intranasally with 200 focus-forming units of ANDV (Chile-9717869) and administered mAbs (MIB22 + JL16) directed against ANDV glycoprotein (*11*) twice by the intraperitoneal route as a cocktail therapy. The study comprised 2 sets of experiments addressing treatment at midstage and late-stage disease. Both sets included control and treatment groups. The treatment group (n = 18) received an injection of the 2 mAbs (25 mg/kg of each mAb), and the control group (n = 18) received an isotype control mAb (50 mg/kg). To determine differences in ANDV replication, we euthanized 6 animals of each group at a predetermined time point (10 days postinfection [dpi]) and kept 12 animals in each group to monitor survival.

**Figure 1:**
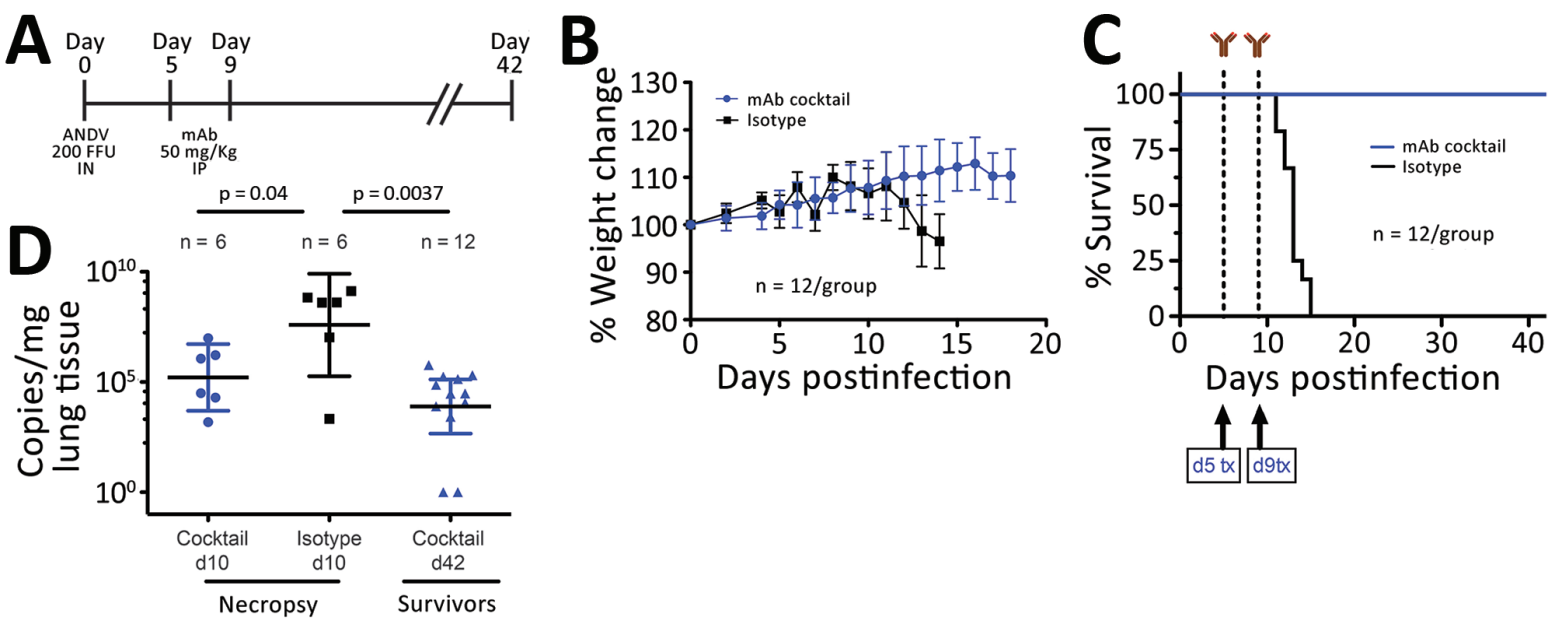
In vivo efficacy of a cocktail of human mAbs specific for ANDV glycoprotein administered at days 5 and 9 postinfection in the Syrian hamster model of hantavirus cardiopulmonary syndrome. A) Syrian hamsters were inoculated intranasally with 200 FFU of ANDV and then administered intraperitoneally a cocktail of mAb (JL16 + MIB22, 25 mg/kg each) or isotype control (50 mg/kg) on day 5 and day 9 postinfection. B) Percentage of weight change monitored until 18 days postinfection, represented as the average per group. Error bars indicate 95% CIs. C) Statistical evaluation of survival by group. Survival was evaluated at p<0.0001 by Mantel-Cox log-rank test using GraphPad Prism (GraphPad Software, Inc., https://www.graphpad.com); p<0.05 was significant. d5 tx and d9 tx indicate treatment schedule (5 and 9 days postinfection). D) ANDV RNA copies per milligram of lung tissue on day 10 postinfection (d10) and 42 days postinfection (d42). Samples were compared to a standard curve using an in vitro transcribed ANDV RNA fragment of known small segment copy number. p values by unpaired t-test using GraphPad Prism. Symbols indicate geometric means; horizontal line indicates median; error bars indicate 95% CIs. ANDV, Andes virus; FFU, focus-forming units; mAbs, monoclonal antibodies.

First, we determined the efficacy of the mAb cocktail administered at 5 and 9 days dpi (5+9) to a treatment group (n = 12), compared with a control group (n = 12) treated with isotype antibody (Figure 1, panel A). The control animals had a median time to death of 13 dpi, as previously observed (*12*). We monitored the animals and performed weight and physical examinations daily until euthanasia or until no clinical signs of illness were observed for 2 consecutive days (treatment group; day 18) (Figure 1, panel B). Human mAb cocktail treatment was well tolerated and resulted in 100% protection from ANDV challenge (Figure 1, panel C; p<0.0001). We necropsied 6 animals at 10 dpi to determine lung viral loads by quantitative reverse transcription PCR as previously described (*11*). Results showed that the group treated with the mAb cocktail had significantly reduced lung viral RNA copy numbers compared with those for the isotype control group (Figure 1, panel D; p = 0.04). We also measured viral RNA in animals at 42 dpi. Although we detected viral RNA in lung tissue, the amount of viral RNA was significantly lower compared with that in animals necropsied at 10 dpi (Figure 1, panel D; p = 0.0037); this finding resembles the pathology seen in humans in which viral RNA can be detectable even months after resolution of acute infection. Although detectable viral RNA does not translate necessarily into infectious virus, this finding suggests that disease manifestation and outcome are not directly associated with the presence of viral RNA (*14*).

**Figure 2:**
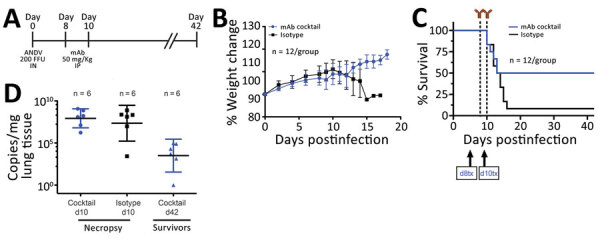
In vivo efficacy of a cocktail of human mAbs specific for ANDV glycoprotein administered at days 8 and 10 postinfection in the Syrian hamster model of hantavirus cardiopulmonary syndrome. A) Syrian hamsters were inoculated intranasally with 200 FFU of ANDV and then administered intraperitoneally a cocktail of mAbs (JL16 + MIB22; 25 mg/kg each) or isotype control (50 mg/kg) on day 8 and day 10 postinfection. B) Percentage of weight change monitored until 18 days postinfection, represented as the average per group. Error bars indicate 95% CIs. C) Statistical evaluation of survival by group. Survival was evaluated at p = 0.123 by Mantel-Cox log-rank test using GraphPad Prism (GraphPad Software, Inc., https://www.graphpad.com); p<0.05 was significant. d8 tx and d10 tx indicate treatment schedule (8 and 10 days postinfection). D) ANDV RNA copies per milligram of lung tissue on day 10 (d10) and day 42 (d42) postinfection. Samples were compared to a standard curve using an in vitro transcribed ANDV RNA fragment of known small segment copy number. Symbols indicate geometric means; horizontal line indicates median; error bars indicate 95% CIs. ANDV, Andes virus; FFU, focus-forming units; mAbs, monoclonal antibodies.

To further assess the efficacy of the mAb cocktail against ANDV, we evaluated the mAb cocktail treatment at an advanced stage of ANDV infection by treating the animals at 8 and 10 dpi (8+10) (Figure 2, panel A) (n = 12). We monitored these animals and recorded weight changes (Figure 2, panel B). Eleven of the 12 control-group animals treated with isotype antibody succumbed to disease or met the euthanasia criteria. The single surviving control animal showed clinical signs of disease that scored just below the euthanasia criteria, which include signs of moribundity: ataxia, reluctancy to move, bleeding from any orifice, tachypnea, or paralysis. The isotype control group showed a median survival time of 12 dpi. In the mAb cocktail treatment group, 6 hamsters survived; although our results (p = 0.123) were not statistically significant (at a p<0.05 level), the mAb cocktail showed 50% efficacy when administered at the time points of 8 and 10 dpi (Figure 2, panel C). As before, we necropsied 6 animals at 10 dpi to determine ANDV RNA loads in the lungs. We observed no significant differences in viral RNA loads between the mAb cocktail–treated group and control groups (Figure 2, panel D). Finally, the 6 surviving hamsters that were necropsied at 42 dpi showed a decrease in lung viral loads, compared with the group that was necropsied at 10 dpi (Figure 2, panel D). Ultimately, we measured the concentration of human IgG in serum samples from animals necropsied at 10 dpi (Appendix Figure 1).

## Conclusions

Our results demonstrate that the human mAb cocktail containing JL16 and MIB22 is able to completely or partially protect Syrian hamsters against lethal ANDV challenge when administered as a 2-dose regimen at midstage (5 and 9 dpi) and late-stage (8 and 10 dpi) disease. To date, the Syrian hamster is the only ANDV preclinical disease model that is appropriate to evaluate the efficacy of therapeutic and prophylactic countermeasures. Thus, our antibody cocktail study meets the Food and Drug Administration animal rule, which requires evidence of efficacy of a countermeasure in a well-established and evaluated animal model indicating potential benefit for human use (*13*). Our results highlight the potential of recombinant human mAbs JL16 and MIB22 to be used as postexposure countermeasure, even at later stages of infection. This finding is supported by a previous study (*15*) showing that immune plasma containing neutralizing antibodies decreases the case-fatality rate in HCPS patients who had mild to severe disease at hospitalization, highlighting the potential of human antibodies to alter the clinical outcome.

Fine-tuning the dosing and more effective combinations of mAbs will likely extend the therapeutic window even further. However, the 100% midstage and 50% late-stage protection observed in these experiments are very encouraging in the endeavor to find a specific treatment for human HCPS caused by ANDV infection.

Appendix Additional information about human monoclonal antibody treatment for Andes virus infection in Syrian hamsters.

## References

[1] Figueiredo LT, Souza WM, Ferrés M, Enria DA. Hantaviruses and cardiopulmonary syndrome in South America. Virus Res. 2014;187:43–54. 10.1016/j.virusres.2014.01.015 24508343

[2] Martinez VP, Bellomo C, San Juan J, Pinna D, Forlenza R, Elder M, et al. Person-to-person transmission of Andes virus. Emerg Infect Dis. 2005;11:1848–53. 10.3201/eid1112.050501 16485469 PMC3367635

[3] Padula PJ, Edelstein A, Miguel SD, López NM, Rossi CM, Rabinovich RD. Hantavirus pulmonary syndrome outbreak in Argentina: molecular evidence for person-to-person transmission of Andes virus. Virology. 1998;241:323–30. 10.1006/viro.1997.8976 9499807

[4] Ferrés M, Vial P, Marco C, Yanez L, Godoy P, Castillo C, et al.; Andes Virus Household Contacts Study Group. Prospective evaluation of household contacts of persons with hantavirus cardiopulmonary syndrome in chile. J Infect Dis. 2007;195:1563–71. 10.1086/516786 17471425

[5] Martínez VP, Di Paola N, Alonso DO, Pérez-Sautu U, Bellomo CM, Iglesias AA, et al. “Super-spreaders” and person-to-person transmission of Andes virus in Argentina. N Engl J Med. 2020;383:2230–41. 10.1056/NEJMoa2009040 33264545

[6] Jonsson CB, Hooper J, Mertz G. Treatment of hantavirus pulmonary syndrome. Antiviral Res. 2008;78:162–9. 10.1016/j.antiviral.2007.10.012 18093668 PMC2810485

[7] Valdivieso F, Vial P, Ferres M, Ye C, Goade D, Cuiza A, et al. Neutralizing antibodies in survivors of Sin Nombre and Andes hantavirus infection. Emerg Infect Dis. 2006;12:166–8. 10.3201/eid1201.050930 16494739 PMC3291396

[8] MacNeil A, Comer JA, Ksiazek TG, Rollin PE. Sin Nombre virus-specific immunoglobulin M and G kinetics in hantavirus pulmonary syndrome and the role played by serologic responses in predicting disease outcome. J Infect Dis. 2010;202:242–6. 10.1086/653482 20521946

[9] Hooper JW, Brocato RL, Kwilas SA, Hammerbeck CD, Josleyn MD, Royals M, et al. DNA vaccine-derived human IgG produced in transchromosomal bovines protect in lethal models of hantavirus pulmonary syndrome. Sci Transl Med. 2014;6:264ra162. 10.1126/scitranslmed.3010082 25429055

[10] Duehr J, McMahon M, Williamson B, Amanat F, Durbin A, Hawman DW, et al. Neutralizing monoclonal antibodies against the Gn and the Gc of the Andes virus glycoprotein spike complex protect from virus challenge in a preclinical hamster model. MBio. 2020;11:e00028–20. 10.1128/mBio.00028-20 32209676 PMC7157512

[11] Garrido JL, Prescott J, Calvo M, Bravo F, Alvarez R, Salas A, et al. Two recombinant human monoclonal antibodies that protect against lethal Andes hantavirus infection in vivo. Sci Transl Med. 2018;10:eaat6420. 10.1126/scitranslmed.aat6420 30463919 PMC11073648

[12] Safronetz D, Zivcec M, Lacasse R, Feldmann F, Rosenke R, Long D, et al. Pathogenesis and host response in Syrian hamsters following intranasal infection with Andes virus. PLoS Pathog. 2011;7:e1002426. 10.1371/journal.ppat.1002426 22194683 PMC3240607

[13] Safronetz D, Ebihara H, Feldmann H, Hooper JW. The Syrian hamster model of hantavirus pulmonary syndrome. Antiviral Res. 2012;95:282–92. 10.1016/j.antiviral.2012.06.002 22705798 PMC3425723

[14] Kuenzli AB, Marschall J, Schefold JC, Schafer M, Engler OB, Ackermann-Gäumann R, et al. Hantavirus cardiopulmonary syndrome due to imported Andes hantavirus infection in Switzerland: a multidisciplinary challenge, two cases and a literature review. Clin Infect Dis. 2018;67:1788–95. 10.1093/cid/ciy443 30084955 PMC6233683

[15] Vial PA, Valdivieso F, Calvo M, Rioseco ML, Riquelme R, Araneda A, et al.; Hantavirus Study Group in Chile. A non-randomized multicentre trial of human immune plasma for treatment of hantavirus cardiopulmonary syndrome caused by Andes virus. Antivir Ther. 2015;20:377–86. 10.3851/IMP2875 25316807

