# Supplementary material for: Therapeutic Efficacy of Human Monoclonal Antibodies against Andes Virus Infection in Syrian Hamsters
Source: Emerg Infect Dis. 2021 Oct;27(10):2707–10. doi: 10.3201/eid2710.210735 (PMC8462347; doi:10.3201/eid2710.210735)
Supplement: Appendix — Additional information about human monoclonal antibody treatment for Andes virus infection in Syrian hamsters. [file 21-0735-Techapp-s1.pdf]

Article DOI: https://doi.org/10.3201/eid2710.210735

Therapeutic Efficacy of Human Monoclonal Antibodies against Andes Virus Infection in Syrian Hamsters

Appendix

We measured the concentration of human IgG antibodies in serum samples from necropsied hamsters using the IgG human SimpleStep ELISA Kit (Abcam, https://www.abcam.com) according to the manufacturer´s instructions. Hamsters in the necropsied 5+9 days postinfection (dpi) group received 2 doses of isotype control (50mg/kg) or monoclonal antibody (mAb) cocktail (JL16 + MIB22, 25mg/kg of each mAb) at day 5 and day 9 postinfection. In contrast, the necropsied hamsters from the 8+10 dpi group received only 1 dose of isotype control (50mg/kg) or 1 dose of mAb cocktail (JL16 + MIB22, 25mg/kg of each mAb) at 8 dpi, then were necropsied at 10 dpi. Accordingly, to the mAb administration doses, we observed higher levels of human IgG Abs in the necropsied 5+9 dpi group (Appendix Figure).

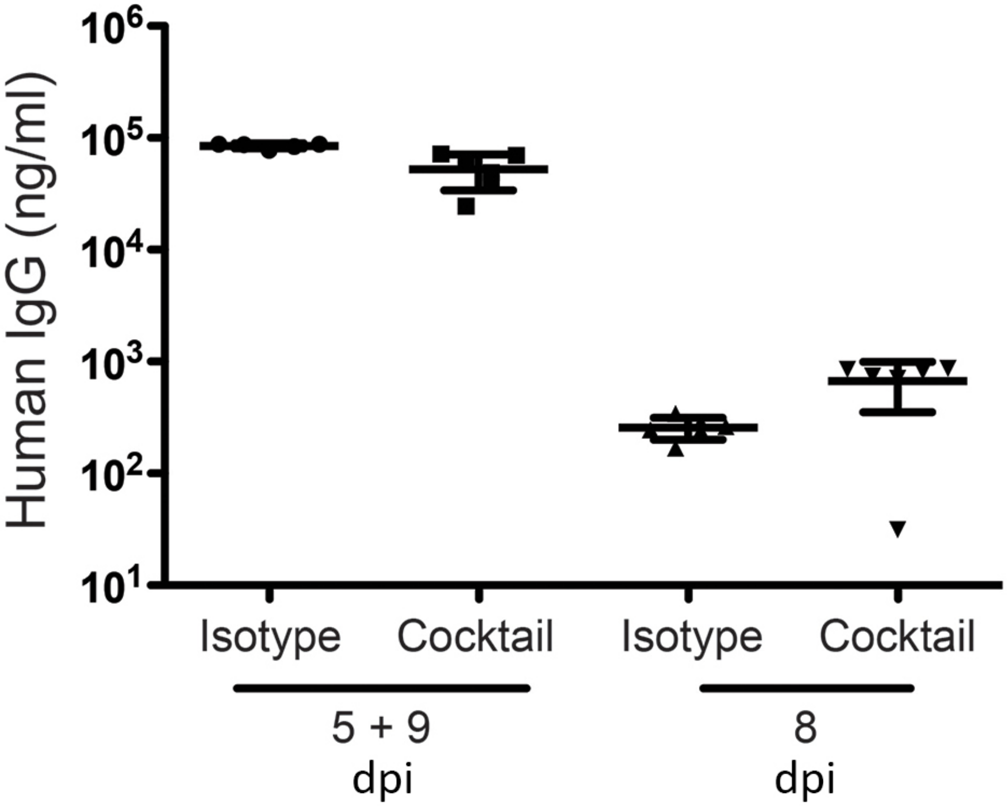

Appendix Figure. Measurement of human IgG antibodies in necropsied hamsters at day 10 postinfection. Serum samples from necropsied animals on day 10 postinfection were used to measure the concentration of human IgG antibodies by ELISA. The left side of the graph shows concentration (ng/mL) of human IgG from serum of hamsters that received isotype control (50 mg/kg) or a cocktail of mAb (JL16 + MIB22; 25 mg/kg each) administered at day 5 and day 9 postinfection (5+9 dpi). The right sideshows concentration of human IgG from hamsters that received isotype control (50 mg/kg) or a cocktail mAb (JL16 + MIB22; 25 mg/kg each) administered at day 8 postinfection (8 dpi). Dots indicate mean value and lines indicate SD.
